# COVID-19 Pandemic: Group Testing

**DOI:** 10.3389/fmed.2020.00522

**Published:** 2020-08-18

**Authors:** Ozkan Ufuk Nalbantoglu, Aycan Gundogdu

**Affiliations:** ^1^Department of Computer Engineering, Erciyes University, Kayseri, Turkey; ^2^Genome and Stem Cell Center (GenKok), Erciyes University, Kayseri, Turkey; ^3^Department of Microbiology and Clinical Microbiology, Erciyes University, Kayseri, Turkey

**Keywords:** COVID-19, pandemia, molecular diagnostics, infection control, group testing

## Introduction

The COVID-19 outbreak has revealed modern society to be negligently unprepared for a pandemic. Despite all the advancements in modern healthcare, our response has been that of a century ago. Back then, social distancing was the main mitigation strategy, and convalescent sera was the treatment option. In addition to that, today we have molecular (i.e., PCR) and serological (i.e., IgC/IgM) tests for diagnostics and ventilators for treatment. Given the pace of scientific and technological development in the last century, this pattern implies the lack of translating scientific knowledge to applications.

Leaving the therapeutics out of scope, we would like to comment on the diagnostics perspective from the early stages: infection control via preventive diagnostics. The most natural question to ask is “Was the pandemic inevitable?” This is a tough question to answer. There are strong arguments supporting both answers. On one hand, epidemiological simulations considering the contemporary aviation schedules have arrived at a significant conclusion: it is highly probable that an outbreak of such parameters would have ended up evolving into a pandemic (1). It is indeed hard to reject the notion that SARS-CoV-2, with its relatively long transmissible incubation period, could easily travel and cover the human habitat, and it would be impossible to trace with conventional measures. The mainstream approach to infection control during the global spread has been scanning potential carriers using symptomatic signals (e.g., thermal cameras, thermometer checks, travel questionnaires, etc.) at connecting hubs. However, these preventative measures were shown to be ineffective in eliminating the spread of COVID-19. The main factor why the preventive measures fell short might be the appearance of excessive numbers of asymptomatic/presymptomatic carriers, which are difficult to detect. An unbiased estimation of the ratio of asymptomatic or presymptomatic spreaders might be difficult to assess. Statistics from small- or medium-sized cohorts and case studies indicate that they might be as abundant as 10–50% of the total number of infections (2, 3). These ratios hold special importance regarding the case studies, showing that asymptomatic/presymptomatic carriers are likely to infect their contacts (4, 5).

The counter-argument claiming that a pandemic in the contemporary world would have been preventable relies on an extensive use of modern digital technology. The idea is a working communication infrastructure as an early warning system. It is believed that such a system could enable control of the epidemic at initial phases. This optimism perhaps stems from the early detection success of The Global Public Health Intelligence Network (GPHIN) (6) during the first SARS-CoV-1 outbreak. Previously, it was believed that utilizing cellular networks would be an invaluable non-biotechnological opportunity for early detection and response. Note that this was even before the emergence of mobile technologies, big data, widespread social networks, and the tremendous advances in artificial intelligence fields. The last decade had been a time of blooming opinions and futuristic depictions of how technology and society is transforming into a new and data-driven paradigm. Shifting from the diagnostic care of twentieth century to the preventive strategies of twenty-first century for emerging infectious diseases was obviously no exception. It was expected that big data analytics could be the key to rapid detection and early prevention of the next pandemic (7–9).

## Digital Technology and Data Science in Epidemiology

As of February 2020, almost the half of the world voluntarily carries GPS tracking devices (i.e., smartphones), which can record the mobility of masses^1^. A vast majority of the transmission from ground zero, patient one, and day 1 to the current terminal points sits unexplored in web servers both as transmission networks and as spatial distributions. Nevertheless, falling short of early detection, the technology field has reacted with a great effort to fight against the pandemic. In fact, an artificial intelligence (AI)-backed outbreak risk estimator warned of the Wuhan outbreak, preparing even before the WHO and CDC (10). Mobile tracking of possible infections has been extensively used, firstly by South Korea and Singapore (11), and it has rapidly become a widespread technological help used by several nations (12). In addition, the use of data science and big data analytics from rich information sources appears to be on track. Simple digital surveys to locate infection clusters (13) and monitoring surveillance using online data sources (14) were adopted as common practices. Diagnostics using AI-backed biomedical signal processing on medical imaging emerged with practical applications. Deep learning on computerized tomography scans aims to remove the burden on the physicians overwhelmed by the explosion of cases (14, 15). Digital technology was not enough alone to prevent a pandemic; however, it is plausible that it is transforming into strong tools with which to fight and perhaps mitigate it. It might not be possible to conclude whether the state of the art can prevent or control pandemics in the mist of crisis, and there is little data yet to prove this. However, it is agreed that digital technology and data science should be destined to be an integral part of epidemiology in post-COVID-19 practice (16).

## Molecular Diagnosis of COVID-19

Besides being caught short in several fields from public health measures to digital technology, we advocate the claim that the greatest aforementioned scientific translation is in the field of molecular diagnostics. The popular view sets a premium on testing, assessing which is the single most effective weapon with which to track, explore, and isolate the transmission clusters^2^. In fact, testing strategies of different nations have interestingly validated the importance of testing as a preventative diagnostics strategy. The supporting data assessed by epidemic curve characteristics showed the effectiveness of mass testing regimes (17). Motivated by the revealing data, public health decision makers all around the world are trying to switch to extensive testing setups in order to reduce the infection transmission as much as possible. At this point, it is worth questioning the testing routine adopted by the global community. The mainstream molecular method of COVID-19 diagnosis is PCR-based amplicon detection (RT-qPCR in a practical set up) of SARS-CoV-2 genetic material. With widespread infrastructure and routine experience, this seems to be a natural and feasible solution. However, considering the capacity and current scalability options of PCR testing, it is uncertain whether this is the “extensive testing” scenario we are dealing with. By early April 2020, the total amount of tests conducted were in the millions band^3^. The United States, performing the greatest number of daily tests with more than 100,000 tests/day^4^, is now seeing a surge in testing capacity. As the epidemic curve is steepened, it is very likely that the current regime is underperforming. While the current approach is a peacetime (i.e., endemic dynamics) convention, we are in wartime (i.e., pandemic dynamics), which requires its own unique measures.

Along with the technical scalibility issues of current testing conventions, it should also be taken into consideration that waiting to initiate and ramp up the testing availability contributes to the development of steep epidemic curves. These factors are heavily reliant on the differing response policies of governments (18, 19), complex legal oversights for the eligibility to test (20), and technical and economical unpreparedness, especially in third-world countries (21). Regardless of the state of the art molecular testing, the related social issues would have been a significant obstacle to employ extensive testing.

## How Far Can we go in Testing With the Available Resources and What Would we Face?

Outbreak simulations imply that even an imperfect detection and isolation at population levels might be enough to control the COVID-19 outbreak (22). It is difficult to assess whether there are stability breakpoints after which social isolation remains the single most effective measure to reduce risk of spread. However, it can be hypothesized that widespread scale testing—enough to trace more than 70% of contacts—at early arrival phases will be very effective in controlling the outbreaks. Considering that the first wave of outbreaks might have not hit certain societies, and resurgence will still be a great risk globally; more aggressive large-scale testing techniques need to be a priority of molecular microbiology. Furthermore, even in the late epidemic phases, large-scale testing would be a dampening factor flattening the epidemic curves.

As per wartime resources, we do not refer to novel molecular techniques or groundbreaking early-level technologies but very common conventions: RT-qPCR and next-generation sequencing. It could be possible to scale up the testing capacity at orders of magnitude, introducing only simple procedures on well-known daily lab routines. Firstly, considering that the popular biotechnological subject of the last 15 years has abruptly disappeared from the radar, the scientific society had been praising the high-throughput capability of next-generation sequencing. To date, the attempts to use NGS have been mainly on the sequence analysis of SARS-CoV-2^5^ (23, 24), and it has not become a common procedure of testing. The underlying reason for this might be the fact that multiplexing and barcoding preferences are not designed for extreme sample numbers. However, theoretically, a single Illumina sequencer can, for example, cover the SARS-CoV-2 genome 12 billion times in a 24-h run^6^; hundreds of thousands if not millions of samples could be tested in a single spot. That could sound like overestimation, neglecting several practical limitations, but feasible proposals with impressive capacity offerings exist^7^. Released the protocols for a massively parallel COVID-19 diagnostic assay enabling simultaneous testing of 19,200 patient samples. The suggested assay includes a clever tweak in which a large number of barcodes are integrated to a reverse transcription step that enables large-scale testing in a single PCR and sequencing run. It is possible to design multitudes of such laboratory procedures that numerous NGS laboratories are capable of adopting and applying in the blink of an eye. Transferring NGS superpower to the COVID-19 testing arsenal would not only remove the burden from veteran and surging PCR technology, but it would also bring the possibility of mass testing one step closer.

## Population Level Scanning for Covid-19

A second opportunity we have been overlooking is not as visible as high-throughput sequencing, but it is an old, well-known wartime tussle invented to exploit limited resources: group testing. Back in the 1940s, the need for screening US army recruits for syphilis arose. As collecting blood samples and performing a single Wassermann test for each man appeared to be quite resource demanding in the circumstances of World War II, pooling blood samples and performing group tests was observed to be quite effective since the disease was relatively rare. Later on, group testing has become a popular topic in the information theory field, enabling orders of magnitude saving from the test numbers while being able to pinpoint sparse positives accurately (25). Similarly, the attractiveness of recovering sparse signals from a small number of measurements led, in the mid-2000s, to the birth of an entire research area called compressive sampling (compressed sensing) in the signal processing field around (26). Compressive sampling ideas converge into group testing for special settings where sampling matrices are binary (pooling) designs. Several theoretical results (27) and practical applications (28) have been reported, and, from a computational point of view, it can be annotated as a mature field. There have been few studies investigating the group testing opportunities in genotyping (29), and it was not a major point of attraction for molecular diagnostics, perhaps because demand was not particularly high. The notion of “a single specimen per test reaction” is now by default synonymous with diagnostic testing. On the other hand, compressing a very large number of tests in random/structured pools (e.g., around 40 to 120 samples per pooling tube) and conducting relatively small numbers of group tests that are decodable to original results is a tempting idea for the purpose of allocating resources efficiently. There are convincing preliminary results showing that pooling samples in feasible ranges would not attenuate the positive signals to undetectable levels in RT-qPCR (30). Similarly, given enough sequencing depth, detectability could be conserved in NGS testing. In fact, elemental ideas of simple group testing are blooming^8^ (31– 33). Sinnott-Armstrong et al. (28) proposed that a simple grouping scheme pooling on rows and columns of well-plates could increase the testing rates to around 4.5- and 7-fold for 96-well plate and 384-well plate applications, respectively, for 1% prevalence of positive cases. This scheme could achieve up to 9.5-fold increase (384-well plate setting) at a testing rate of 0.1% prevalence of positive cases. It should be acknowledged that this valuable boost does not explore the theoretical and practically achievable rates of compressive sampling capabilities. While theoretically perfect, reconstruction of original test results available with not much more than k log_2_(N/k) measurements (25), where N is the number of samples and k is the number of positive cases, with the use of modern decoding algorithms, the achievable rates are close to the theoretical bounds. This means a 10- to 20-fold rate increase for a 1–0.1% prevalence band is possible with more sophisticated pooling schemes and decoding algorithms. In fact, allowing for more than one round of testing, namely, adaptive testing, instead of one-shot recovery of results, can provide even more efficient outcomes. Especially for low prevalence regimes (i.e., P <1/K^2^), N (2P + (1-2P)/K) measurements set a lower bound on the number of required tests, where P is the prevalence and K the limit of the pool size (34). This implies almost a couple of tests per a positive sample and a single test per pool—a very efficient scheme with large pools. Recently, Shental et al. (35) sampled 48 pools out of a 384 well-plate by way of Reed-Solomon coding and showed an 8X efficiency gain around the band of 1% prevalence in a realistic laboratory setting. In fact, simulations showed that up to 60X expansion in testing capacity is available at around 2–3% of the prevalence band (36). This result might be an implication that large-scale contact tracing might be possible at early forming clusters. A further fascinating result we can draw from the compressive sampling field is that, as the number of samples increase and the prevalence decreases, the sampling efficiency scales up to impressive rates. This phenomenon would result in ultra-throughput testing with a moderate number of actual tests. For example, for the case of sudocodes, at a prevalence of 0.1%, 1 million subjects can be scanned by performing under 10,000 tests (37). We can assume that this scenario realistically fits into the population level testing ambition, in case of early arrival of the pandemic curve. The possible scenarios might be either as extending contact tracing to be able to test greater number of case contacts or as a periodic scan of specific populations such as scans at the level of family, school, classroom, workplace/office, daycare, healthcare workers, and other at-risk groups, staying within the available testing budgets. The opportunity for periodic economical scanning of specific groups could be operationalized as a powerful security measure in the phase of reopening economies. Taking the NGS recruit discussion above into consideration (i.e., tens of thousands of tests can be run on a single sequencer with a single PCR reaction), it can even be proposed that scanning of a million subjects could potentially be conducted in one diagnostic center in a single shot. Of course, this assessment neglects the enormous swab sampling, logistics, and sample preparation aspects. Our sole claim here, however, is that, with the modern molecular diagnostics technology, population level scanning should not be a real bottleneck in outbreak control.

## Conclusion

The COVID-19 pandemic has caught modern society unprepared. Imposed outbreak measures have fallen short of mitigating the evolution of the outbreak into a pandemic. In this opinion article, we discussed whether infection control via preventive diagnostics could be a strong tool in our fight. Currently, digital technology and data science are becoming integral tools with which to help in the control of outbreaks. Although there have been great advancements in molecular technologies, there seems to be a lack of scientific translation in molecular diagnostics. With its surging capacity, RT-qPCR use in COVID-19 diagnosis is underperforming when conducting population-level scans. Despite its grand potential in high-throughput diagnosis, the next-generation sequencing systems have not been deployed sufficiently. Moreover, advanced algorithms to conduct group testing could enable large-scale testing for detecting and isolating infection clusters. Therefore, the scientific community should seek ways to translate invaluable technical expertise to fighting the COVID-19 pandemic, and it should also seek to integrate next-generation tools to contemporary practice. Testing *en masse* might not be as infeasible as it is confined to limited ideas and practices. The availability of detecting and isolating emerging clusters, thus minimizing the infection contacts, could pave the road to avoiding nation-level lockdowns and undetermined periods of quarantine measures. Otherwise, relying on only social distancing will be nothing but failing the “test.”

## Author Contributions

All authors listed have equally made a substantial, direct and intellectual contribution to the work, and approved it for publication.

## Conflict of Interest

The authors declare that the research was conducted in the absence of any commercial or financial relationships that could be construed as a potential conflict of interest.

## Footnotes

^1^https://www.statista.com/statistics/330695/number-of-smartphone-users-worldwide/

^2^https://www.who.int/docs/default-source/coronaviruse/transcripts/who-transcript-emergencies-coronavirus-press-conference-full13mar2020848 c48d2065143bd8d07a1647c863d6b.pdf?sfvrsn=23dd0b04__2

^3^https://ourworldindata.org/covid-testing

^4^https://covidtracking.com/data/

^5^https://nanoporetech.com/about-us/news/uk-creates-covid-19-genome-sequencing-alliance-large-scale-analysis-virus-oxford

^6^https://www.illumina.com/content/dam/illumina-marketing/documents/products/datasheets/novaseq-6000-system-specification-sheet-770-2016-025.pdf

^7^https://docs.google.com/document/d/1kP2w__uTMSep2UxTCOnUhh1TMCjWvHEY0sUUpkJHPYV4

^8^https://www.tse-fr.eu/sites/default/files/TSE/documents/doc/by/gollier/group_testing.pdf

## References

[1] Sato AH Ito I Sawai H Iwata K An epidemic simulation with a delayed stochastic SIR model based on international socioeconomic-technological databases. in 2015 IEEE International Conference on Big Data (Big Data). Santa Clara, CA: IEEE (2015). p. 2732–41.

[2] Mizumoto K Kagaya K Zarebski A Chowell G. Estimating the asymptomatic proportion of coronavirus disease 2019 (COVID-19) cases on board the Diamond Princess cruise ship, Yokohama, Japan, 2020. Euro Surveill. (2020) 25:2000180. 10.2807/1560-7917.ES.2020.25.10.2000180 32183930 PMC7078829

[3] Nishiura H Kobayashi T Miyama T Suzuki A Jung S Hayashi K. Estimation of the asymptomatic ratio of novel coronavirus infections (COVID-19). Int J Infect Dis. (2020) 94:154–5. 10.1016/j.ijid.2020.03.020 32179137 PMC7270890

[4] Bai Y Yao L Wei T Tian F Jin DY Chen L. Presumed asymptomatic carrier transmission of COVID-19. JAMA. (2020) 323:1406–7. 10.1001/jama.2020.2565 32083643 PMC7042844

[5] Gandhi M Yokoe DS Havlir DV. Asymptomatic transmission, the Achilles' heel of current strategies to control COVID-19. N Engl J Med. (2020) 382:2158–60. 10.1056/NEJMe2009758 32329972 PMC7200054

[6] Mykhalovskiy E Weir L. The global public health intelligence network and early warning outbreak detection. Can J Public Health. (2006) 97:42–4. 10.1007/BF03405213 16512327 PMC6976220

[7] Astill J Dara RA Fraser ED Sharif S Detecting and predicting emerging disease in poultry with the implementation of new technologies and big data: a focus on Avian Influenza Virus. Front Vet Sci. (2018) 5:263 10.3389/fvets.2018.00263 30425995 PMC6218608

[8] USAID (U,.S. Agency for International Development) Emerging Pandemic Threats (2016). Available online at: https://www.usaid.gov/news-information/fact-sheets/emerging-pandemic-threats-program (accessed June 2, 2016).

[9] Dion M Abdel Malik P Mawudeku A. Big data: big data and the global public health intelligence network (GPHIN). Can Commun Dis Rep. (2015) 41:209. 10.14745/ccdr.v41i09a02 29769954 PMC5933838

[10] Inn TL Smart City Technologies Take on COVID-19. Penang: World Health (2020).

[11] Zastrow M. South Korea is reporting intimate details of COVID-19 cases: has it helped? Nature. (2020). 10.1038/d41586-020-00740-y. [Epub ahead of print]. 32203363

[12] Ienca M Vayena E. On the responsible use of digital data to tackle the COVID-19 pandemic. Nat Med. (2020) 26:463–4. 10.1038/s41591-020-0832-5 32284619 PMC7100462

[13] Rossman H Keshet A Shilo S. A framework for identifying regional outbreak and spread of COVID-19 from one-minute population-wide surveys. Nat Med. (2020) 26:634–8. 10.1038/s41591-020-0857-9 32273611 PMC7144578

[14] Ting DSW Carin L Dzau V Wong TY. Digital technology and COVID-19. Nat Med. (2020) 26:459–61. 10.1038/s41591-020-0824-5 32284618 PMC7100489

[15] Shan F Gao Y Wang J Shi W Shi N Han M Lung Infection Quantification of COVID-19 in CT Images with Deep Learning. arXiv preprint arXiv:2003.04655 (2020).

[16] Gozes O Frid-Adar M Greenspan H Browning PD Zhang H Ji W Rapid AI development cycle for the coronavirus (covid-19) pandemic: initial results for automated detection & patient monitoring using deep learning CT image analysis. arXiv preprint arXiv:2003.05037 (2020).

[17] Balilla J Assessment of COVID-19 Mass Testing: The Case of South Korea (2020). Available online at: https://ssrn.com/abstract=3556346

[18] Carter DP May PJ Making sense of the US COVID-19 pandemic response: a policy regime perspective. Admin TheorPraxis. (2020) 42:265–77. 10.1080/10841806.2020.1758991

[19] Shvetsova O Catalano M Chu H Dumond GK Muftuoglu E Ozutemiz H Policy Error and Policy Rescue in COVID-19 Responses in the United States and United Kingdom (2020). Available online at: https://orb.binghamton.edu/working_paper_series/3/

[20] McDermott A. Inner Workings: molecular biologists offer “wartime service” in the effort to test for COVID-19. Proc Natl Acad Sci USA. (2020) 117:9656–9. 10.1073/pnas.2006240117 32295880 PMC7211940

[21] Gilbert M Pullano G Pinotti F Valdano E Poletto C Boëlle P-Y. Preparedness and vulnerability of African countries against importations of COVID-19: a modelling study. Lancet. (2020) 395:871–7. 10.1016/S0140-6736(20)30411-6 32087820 PMC7159277

[22] Hellewell J Abbott S Gimma A Bosse NI Jarvis CI Russell TW. Feasibility of controlling COVID-19 outbreaks by isolation of cases and contacts. Lancet Glob Health. (2020) 8:e488–96. 10.1016/S2214-109X(20)30074-7 32119825 PMC7097845

[23] Andersen KG Rambaut A Lipkin WI Holmes EC Garry RF. The proximal origin of SARS-CoV-2. Nat Med. (2020) 26:450–2. 10.1038/s41591-020-0820-9 32284615 PMC7095063

[24] Sah R Rodriguez-Morales AJ Jha R Chu DKW Gu H Peiris M. Complete genome sequence of a 2019 novel coronavirus (SARS-CoV-2) strain isolated in Nepal. Microbiol Resour Announc. (2020) 9:e00169-20. 10.1128/MRA.00169-20 32165386 PMC7067954

[25] Aldridge M Johnson O Scarlett J Group testing: an information theory perspective. Found Trends Commun Inform Theor. (2019) 15:196–392. 10.1561/0100000099

[26] Candès EJ Wakin MB An introduction to compressive sampling. IEEE Signal Process Magaz. (2008) 25:21–30. 10.1109/MSP.2007.914731

[27] Atia GK Saligrama V Boolean compressed sensing and noisy group testing. IEEE Trans Inform Theor. (2012) 58:1880–901. 10.1109/TIT.2011.2178156

[28] Sinnott-Armstrong N Klein D Hickey B Evaluation of group testing for SARS-CoV-2 RNA. medRxiv [Preprint]. (2020). 10.1101/2020.03.27.20043968

[29] Erlich Y Gordon A Brand M Hannon GJ Mitra PP. Compressed genotyping. IEEE Trans Inform Theor. (2010) 56:706–23. 10.1109/TIT.2009.2037043 21451737 PMC3065185

[30] Yelin I Aharony N Shaer Tamar E Argoetti A Messer E. Evaluation of COVID-19 RT-qPCR test in multi-sample pools. Clin Infect Dis. (2020). 10.1093/cid/ciaa531. [Epub ahead of print]. 32358960 PMC7197588

[31] Shani-Narkiss H Gilday OD Yayon N Landau ID Efficient and practical sample pooling high-throughput PCR diagnosis of COVID-19. medRxiv [Preprint]. (2020). 10.1101/2020.04.06.20052159

[32] de Wolff T Pflüger D Rehme M Heuer J Bittner MI Evaluation of pool-based testing approaches to enable population-wide screening for COVID-19. arXiv preprint arXiv:2004.11851 (2020). 10.1371/journal.pone.0243692 PMC7751875 33347458

[33] Eberhardt JN Breuckmann NP Eberhardt CS. Multi-stage group testing improves efficiency of large-scale COVID-19 screening. J Clin Virol. (2020) 128:104382. 10.1016/j.jcv.2020.104382 32388468 PMC7177109

[34] Csóka E Application-oriented mathematical algorithms for group testing. arXiv preprint arXiv:2005.02388 (2020).

[35] Shental N Levy S Skorniakov S Wuvshet V Shemer-Avni Y Porgador A Efficient high throughputSARS-CoV-2 testing to detect asymptomatic carriers. medRxiv [Preprint]. (2020). 10.1101/2020.04.14.20064618 PMC7485993 32917716

[36] Nalbantoglu O. Group testing performance evaluation for SARS-CoV-2 massive scale screening and testing. BMC Med Res Methodol. (2020) 20:176. 10.1186/s12874-020-01048-1 32615934 PMC7330001

[37] Sarvotham S Baron D Baraniuk RG Sudocodes-fast measurement and reconstruction of sparse signals. In: 2006 IEEE International Symposium on Information Theory. Seattle, WA: IEEE (2006). p. 2804–8.

